# Influence of Backbone Regioregularity on the Optoelectronic and Mechanical Response of Conjugated Polyelectrolyte-Based Hydrogels

**DOI:** 10.1021/acs.jpcb.3c00152

**Published:** 2023-03-07

**Authors:** William R. Hollingsworth, Anna R. Johnston, Manping Jia, Le Luo, Yunjeong Park, Walter Meier, Jack Palmer, Marco Rolandi, Alexander L. Ayzner

**Affiliations:** † Department of Chemistry and Biochemistry, University of California Santa Cruz, Santa Cruz, California 95064, United States; ‡ Electrical and Computer Engineering Department, University of California Santa Cruz, Santa Cruz, California 95064, United States

## Abstract

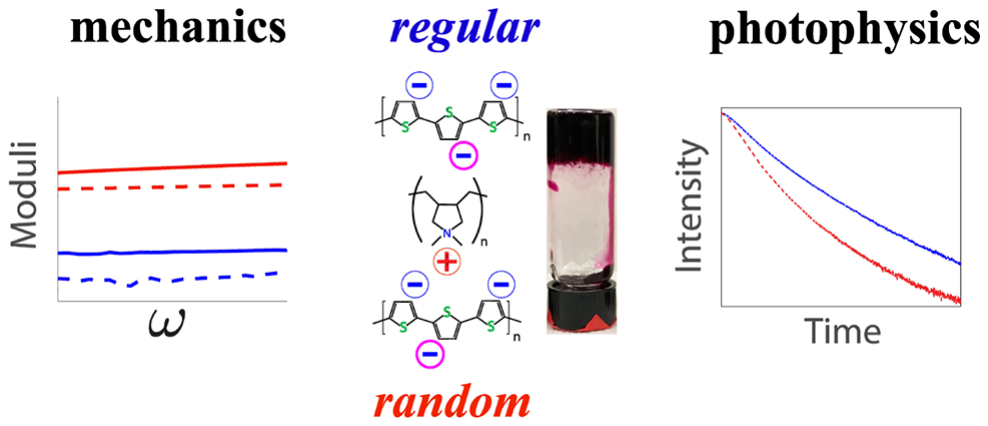

The ability to form robust, optoelectronically responsive, and mechanically tunable hydrogels using facile processing is desirable for sensing, biomedical, and light-harvesting applications. We demonstrate that such a hydrogel can be formed using aqueous complexation between one conjugated and one nonconjugated polyelectrolyte. We show that the rheological properties of the hydrogel can be tuned using the regioregularity of the conjugated polyelectrolyte (CPE) backbone, leading to significantly different mesoscale gel morphologies. We also find that the exciton dynamics in the long-time limit reflect differences in the underlying electronic connectivity of the hydrogels as a function CPE regioregularity. The influence of excess small ions on the hydrogel structure and the exciton dynamics similarly depends on the regioregularity in a significant way. Finally, electrical impedance measurements lead us to infer that these hydrogels can act as mixed ionic/electronic conductors. We believe that such gels possess an attractive combination of physical-chemical properties that can be leveraged in multiple applications.

## 1 Introduction

Optoelectronic hydrogels based on organic semiconductors are promising soft materials for sensing, biomedical, energy-storage, and light-harvesting applications.^1−5^ The potential of environmentally benign aqueous construction and mechanical deformability are also attractive for flexible and stretchable devices,^6^ mixed electron/ion conductors,^7^ and neuromorphic computing.^8^ Water-soluble conjugated polyelectrolytes (CPEs) are particularly intriguing building blocks of such optoelectronic hydrogel materials.^9−17^ The primary reasons for this are their delocalized electronic states, facile processability, synthetic tunability, and their ability to form hierarchical assemblies due to a coexistence of multiple types of intermolecular interactions.^18,19^ CPEs are also intrinsically electronically active, precluding the need to covalently attach dye molecules to a polymeric gelator to gain optoelectronic function.

Yet obstacles in the development and deployment of such materials persist. First, robust and tunable semiconducting hydrogels that are stable under a range of conditions are desirable. Previous work demonstrated that above a critical gelation concentration of ∼10 mg/mL, pure poly(fluorene-*alt*-thiophene) CPEs in aqueous solution and in the absence of excess salt formed hydrogels.^3,20^ Our laboratory found a similar gelation concentration with a related poly(fluorene-*alt*-phenylene) CPE. It was proposed that such gels are composed of cylindrical micelles, with the long axis of the micelle corresponding to the semiconducting backbone direction.^3^ The suggested implication is that charge and exciton transport over long distances should become more efficient in such a cylindrical micelle state. It was found that in the presence of THF, an organic solvent that is miscible with water in all proportions, poly(fluorene-*alt*-thiophene) hydrogels dissolved away. Several questions need to be answered before the attractive properties of CPE-based semiconducting hydrogels can be utilized in soft-materials applications. First, how can the semiconducting hydrogel be made more resistant to dissolution by organic solvents? Doing so is desirable for optoelectronic and biomedical applications, since this would allow one to readily clean the surface of undesirable adsorbents and to possibly disrupt biofouling.

Second, how can one judiciously use the monomer chemical structure and the intermonomer interactions to influence the mechanical and optoelectronic properties of CPE-based hydrogels? It is well-known that both the monomer electronic structure and the intermononer torsional potential affect the chain rigidity. This in turn affects the delocalization of electronic states both along and between conjugated polymer backbones.^21^ Modulation of the polymer regioregularity represents an attractive and possibly straightforward way to manipulate structure–property relationships of semiconducting and conducting hydrogels without the need to alter the chemical structure of the monomer.

There is an additional straighforward means of manipulating the electronic structure of CPE-based hydrogels. We have recently shown that the optoelectronic properties of colloidal gels formed by complexing a nonconjugated polyelectrolyte and an oppositely charged CPE could be altered by introducing additional simple atomic ions.^19,22^ There appears to be a substantial coupling between ionic and electronic degrees of freedom in such systems, which can be further exploited to tune the electronic properties and perhaps the structure of the resulting material. However, the manner in which the regioregularity of the CPE backbone influences the ion-dependent response of these materials remains obscured.

In this work, we interrogate the dependence of the mechanical, photophysical, and structural properties of hydrogels based on the half-conjugated polyelectrolyte complex on the regioregularity of the CPE backbone. We show that complexation between an anionic polythiophene-based CPE and a cationic nonconjugated polyelectrolyte readily forms robust hydrogels that resist dissolution by THF. Using a pair of regioregular and regiorandom CPE stereoisomers, we demonstrate that the mechanical properties of the semiconducting hydrogels in the absence of excess salt depend strongly to the regioregularity of the CPE, while the photophysics is only weakly dependent. Addition of the simple salt KBr alters the loss and storage moduli of both polymers but does not dissociate the interpolyelectrolyte complex or destabilize the gel state. At the same time, we find that the presence of excess salt significantly increases the radiative lifetime and thus the electronic properties of the regiorandom CPE-based hydrogel, but the lifetime of the regioregular stereoisomer undergoes only small changes. Finally, we interrogate the influence of regioregularity on the hydrogel conductivity using impedance spectroscopy and conclude that these hydrogels can act as mixed ionic/electronic conductors.

## 2 Experimental Section

### 2.1 Materials and Hydrogel Preparation

The high-molecular-weight cationic polyelectrolyte poly(diallyldimethylammonium chloride) (PDADMAC) was obtained from Sigma-Aldrich (MW = 400 000–500 000 Da, 1.04 g/mL, 20 wt % in H_2_O). The anionic regioregular CPE poly(butylcarboxythiophene) derivative (regPTAK, MW = 16 000 Da, PDI = 2.2) and regiorandom PTAK (ranPTAK, MW = 8000 Da, PDI = 1.8) were obtained from Rieke Metals. Potassium bromide (KBr, 99.99% purity) and HPLC-grade H_2_O were obtained from Sigma-Aldrich. All chemicals were used as received.

To prepare 1 mL hydrogel samples without added KBr, 334 μL of an aqueous PDAMAC solution (259 μL of HPLC grade water and 75 μL of 208 mg/mL PDADMAC) was added dropwise to 666 μL of 30 mg/mL aqueous PTAK solution while being heating at 70 °C and stirring at 350 rpm. Gels were allowed to heat at this temp and stir-rate for 2 h. Samples were then allowed to cool and set overnight prior to their characterization. To prepare 1 mL samples of hydrogels with 0.5 M KBr, 334 μL of an aqueous PDAMAC/KBr solution (259 μL of 1.93 M KBr with 75 μL of 208 mg/mL PDAMAC stock) was added dropwise to 666 μL of 30 mg/mL aqueous PTAK solution while heating at 70 °C and stirring at 350 rpm. Gels were heated, cooled, allowed to set, and characterized in the same manner described for gels without added KBr.

### 2.2 Confocal Laser Scanning Microscopy

A small portion of sample, roughly 3–4 mm^3^, was placed directly onto a standard microscope slide. A #1.5 coverslip was placed on top of the sample and gentle pressure was applied to distribute the sample evenly on the slide. The edges were sealed using Kapton tape and the sample was imaged through the #1.5 coverslip immediately after mounting. Confocal images were acquired on a Leica SP5 confocal microscope using a 20×/0.75 objective. 488 nm laser was used at 25% power and signal collected between 575 and 775 nm. Scan speed was fixed at 400 Hz and pixel size was 760 nm ± 5 nm for all images. Care was taken to ensure that all images shown are representative and not outliers. Images were processed in ImageJ/FIJI.^1^ Images were processed in ImageJ/FIJI.^1^ Images were processed in ImageJ/FIJI. Images were processed in ImageJ/FIJI.^29^ Processing steps for all selected images included cropping, histogram adjustment, and LUT adjustment.

### 2.3 Rheology

Small-amplitude oscillatory shear (SAOS) measurements were performed on gel samples using strain amplitudes within the linear viscoelastic region and an angular frequency range of 0.1–100 rad/s. All measurements were taken at the Stanford Nano Shared Facility (SNSF): Soft and Hybrid Materials Facility (SMF) using an ARES-G2 strain-controlled rheometer from TA Instruments in strain-controlled oscillatory mode. All experiments were done using a 25 mm cone-and-plate geometry with an angle of 0.1 rad and a truncation gap of 0.05 mm. The temperature was set at 20 °C via a Peltier controller, and a solvent trap was utilized to minimize drying of gels during measurement.

### 2.4 Optical Spectroscopy

To collect UV–vis and PL spectra, a small portion of the hydrogel was sandwiched between standard microscope slides, with the edges sealed by Kapton tape. UV–vis spectra were collected with a 1 nm bandpass with a wavelength increment of 1 nm. PL spectra were collected in front-face detection under laser excitaiton (NKT). PL signal was collected using a Pixis 100 CCD (Princeton Instruments) mounted on a monochromator (Princeton Instruments).

TRPL and time-resolved anisotropy measurements were collected using time-correlated single photon counting on a home-built instrument which has been described previously.^18^ Samples were prepared as described above for steady-state measurements. TRPL measurements were taken at 5 different locations on the sample. For each sample, measurement time was varied to achieve ∼10,000 counts in the main channel. At each position, 10 measurements were collected and averaged. All samples were excited at 450 nm, with emission collected at 675 nm. Deconvolution and fluorescence lifetime analysis were performed in MATLAB using the DecayFit toolbox.

### 2.5 Electrochemical Impedance Spectroscopy

All electrical measurements were performed by using a computer controlled Autolab potentiostat with analytic software Nova 2.0. The measurement was done using a two-terminal device with 100 nm thin film gold as the electrode contact. Each contact is 5 mm × 10 mm and their distance is 50 um. PDMS with an 8 mm diameter hole was attached on the center of two gold electrodes, and the hydrogels were drop casted in the PDMS hole. We measured the impedance of the dry hydrogel after drying in the liophylizer. We measured the impedance of the hydrated hydrogel after exposing the hydrogel to 90% relative humidity for 48 h.

## 3 Results

### 3.1 Rheological Properties

**Scheme 1:**
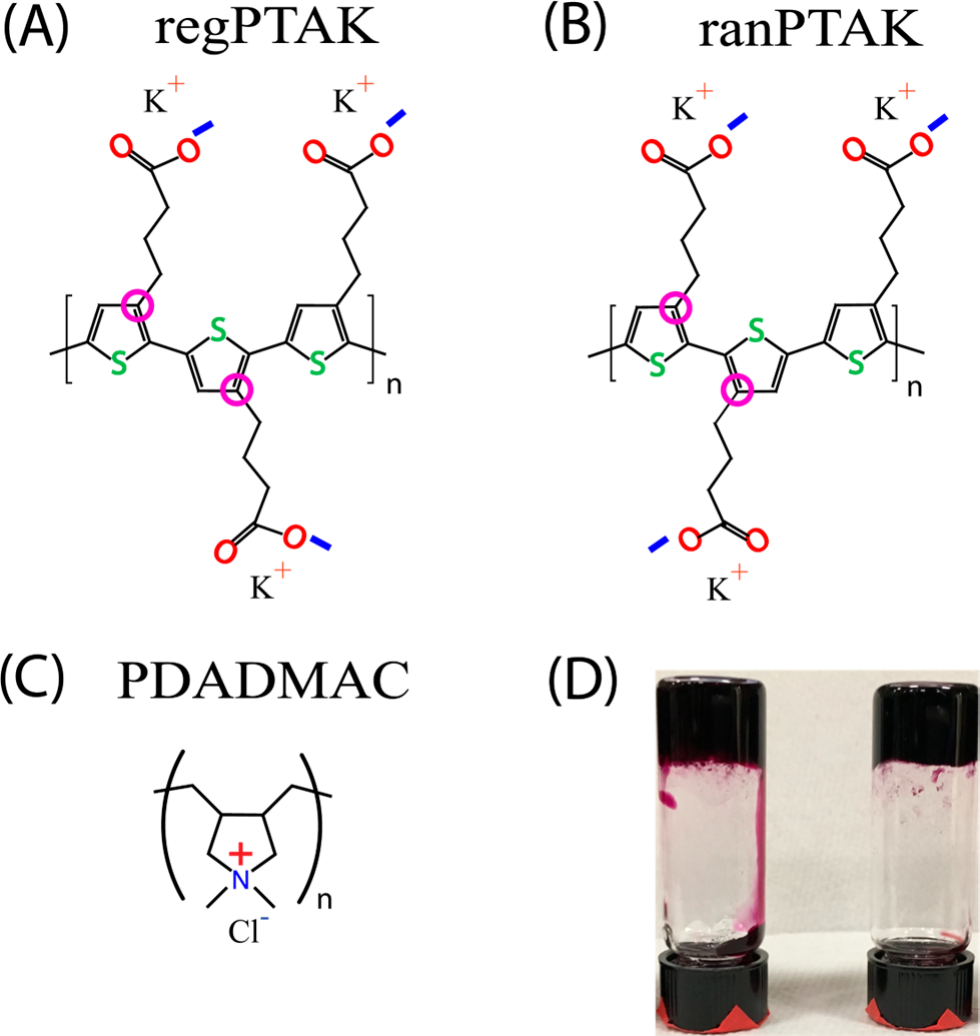
Chemical Structures (A) Regioregular anionic polythiophene derivative, regPTAK; (B) regiorandom anionic polythiophene derivative, ranPTAK; (C) cationic PDADMAC. (D) Photograph showing regPTAK:PDADMAC gels with no added salt (left) and with 0.5 M KBr (right).

Scheme 1 shows the chemical structures of the regioregular (regPTAK, Scheme 1A) and regiorandom (ranPTAK, Scheme 1B) polythiophene-based CPEs, as well as the high-molecular-weight cationic polyelectrolyte PDADMAC (Scheme 1C). Scheme 1D displays photographs of optically dense regPTAK:PDADMAC hydrogels with no excess salt and with 0.5 M KBr. Visual inspection showed that effectively the entire amount of water used to prepare the samples was taken up by the hydrogel volume under the sample preparation conditions used in this paper. There was negligible dilute solution in coexitence with the hydrogel state, both in the presence and absence of KBr. Thus, we believe that the great majority of the added salt is retained within the hydrogel.

**Figure 1:**
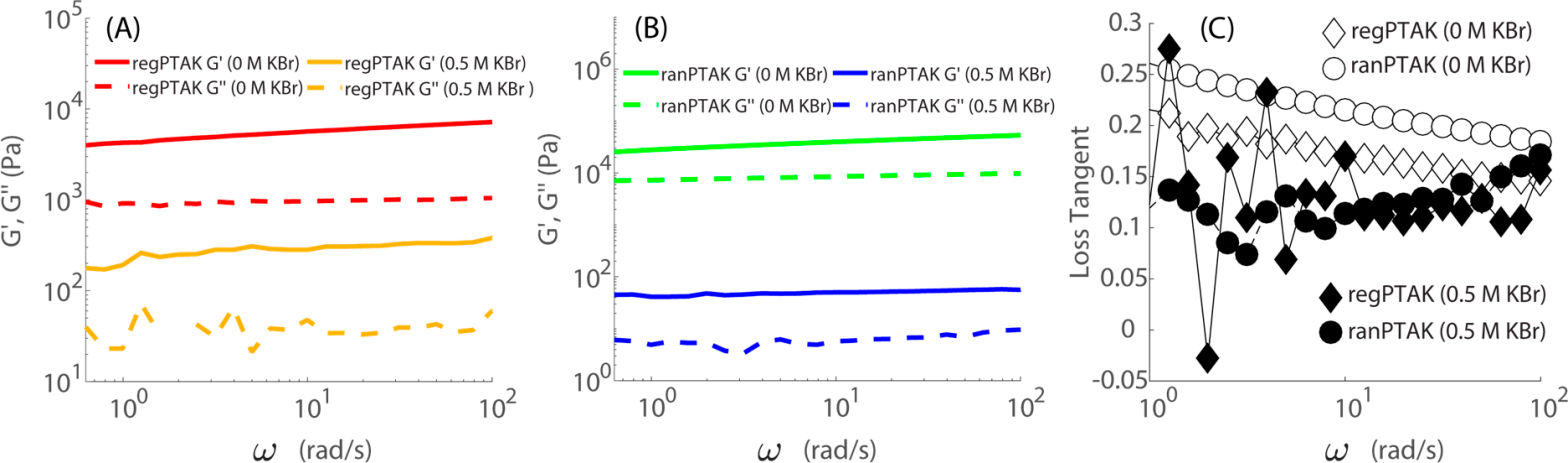
Storage and loss moduli as a function of ω for (A) regPTAK:PDADMAC and (B) ranPTAK:PDADMAC hydrogels. Solid curves correspond to storage moduli and dashed curves to loss moduli. The storage modulus undergoes a large decrease upon addition of 0.5 M KBr for both CPEs, but so does the loss modulus. (C) Loss tangent tan δ for the four gel samples. In the absence of salt, tan δ is larger for ranPTAK than for regPTAK gels, implying that regiorandom backbones promote a more deformable morphology. Both the storage and loss moduli decrease upon addition of KBr for both polymers, leading to a similar tan δ at 0.5 M KBr.

To understand how regioregularity of the anionic CPE influences the mechanical properties of the hydrogels, we performed oscillatory shear measurements as a function of angular oscillation frequency ω. For regPTAK:PDADMAC hydrogels, Figure 1A shows that the storage modulus *G′* dominates over the loss modulus *G*″ both in the absence of excess salt and in the presence of KBr at 0.5 M, and all moduli depend weakly on ω. These observations are consistent with the physical appearance of the gels. We can get a better sense for the difference in deformability of the gels by plotting the loss tangent tan δ(ω) = *G*″/*G*′, where δ is the phase angle. Figure 1C, shows that, in the absence of excess salt, tan δ at 12.5 rad/s is larger for ranPTAK hydrogels than regPTAK hydrogels by 26%. This means that without excess KBr, ranPTAK gels are more deformable. Upon addition of KBr, the gels become less stiff due to a significant decrease in *G′* relative to the no-salt case, with *G′* for ranPTAK samples being ∼80% smaller than that of regPTAK. At the same time *G*″ also decreases, resulting in a mild drop in tan δ. Interestingly, in the presence of excess salt tan δ becomes quite similar for both CPE-based gels.

All hydrogels display shear-thinning behavior. At 12.5 rad/s, the viscosity of the ranPTAK sample in the absence of excess salt is 3.34 × 10^3^ Pa·s, but it drops by nearly 3 orders of magnitude to 4.05 Pa·s in the presence of 0.5 M KBr. For regPTAK at the same frequency, the viscosity with no salt is 3.93 × 10^2^ Pa·s and drops to 24.2 Pa·s upon addition of salt.

It is helpful to compare the storage moduli of these hydrogels with a few gel classes containing organic semiconductors. Naphthalene diimide (NDI) peptide-based hydrogels were found to have *G′* in the 10–200 Pa range depending on the alkyl chain length and the presence of an alkylamine additive.^2^ Poly(fluorene-*alt*-thiophene) (PFT) hydrogels also showed weakly frequency-dependent *G′* in the range of 5–500 Pa depending on [PFT].^3^ Poly(fluorene) and poly(fluorene-*alt*-aryl) viscoelastic fluids and gels made from melts of neutral conjugated polymers containing alkyl side chains were found to have *G′* ∼ 5 × 10^4^ Pa at 10 rad/s.^23^ Finally, perhaps the most relevant reference point is the work done by Shinde et al. Here reversible gels were made by electrostatically cross-linking poly(thiophene)-based CPEs bearing heptylcarboxylate side chains with hexyl diamines.^24^ The backbone and side chain of this CPE are highly analogous to our PTAK derivatives, though the alkyl spacer is substantially longer. It was found that such gels had *G′* ∼ 10^2^ Pa. Our results show that both PTAK stereoisomers complexed with PDADMAC form hydrogels with *G′* that are more than an order of magnitude larger than the NDI and PFT hydrogels and that are similar to those of the neutral poly(fluorene-*alt*-aryl) gels with a large alkyl side chain content.^24^ At 0.5 M KBr, both *G′* and *G*″ of PTAK:PDADMAC hydrogels decrease by a factor of 20 at 10 rad/s to values more comparable to those of NDI and PFT gels.

### 3.2 Steady-State Spectroscopy

How do differences in mechanical properties of regPTAK and ranPTAK hydrogels manifest in their photophysical properties? To answer this question, we first collected steady-state optical density (OD) and photoluminescence (PL) spectra of the hydrogels in the absence and presence of excess KBr. All PL measurements were done via front-face detection on gel samples sandwiched between microscope slides and sealed with Kapton tape to minimize water evaporation during measurements.

**Figure 2:**
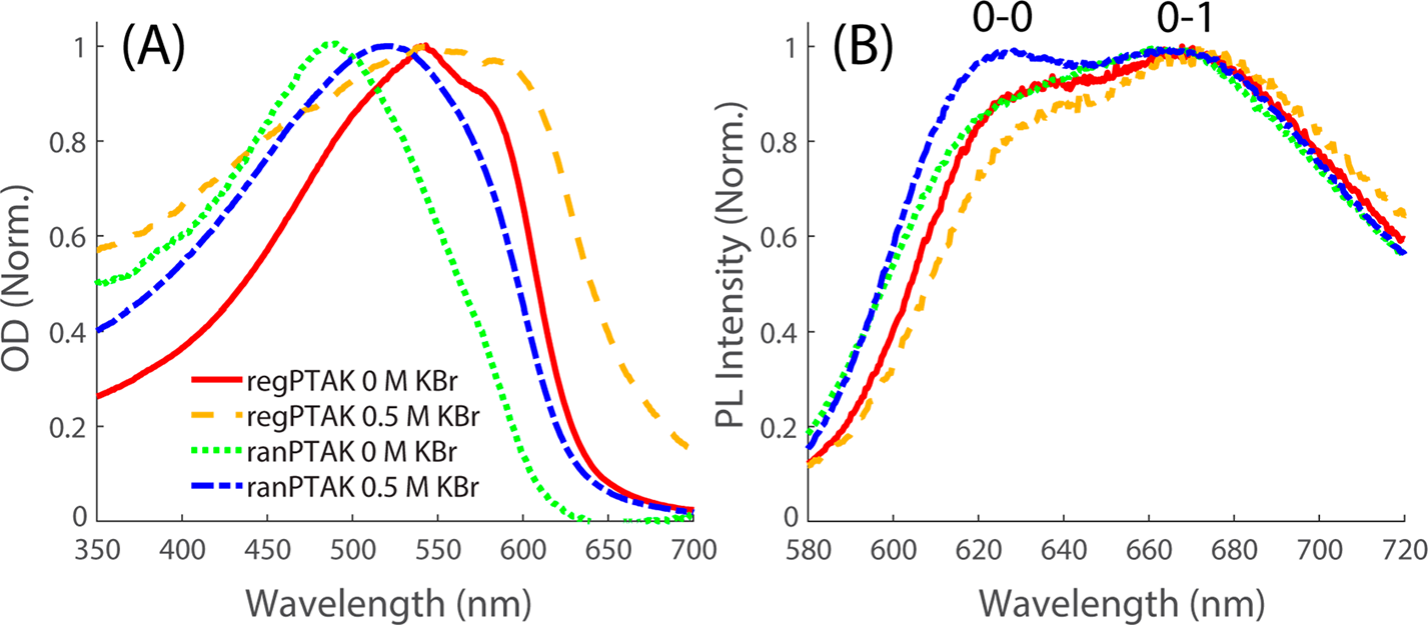
Steady-state OD (A) and PL (B) spectra of PTAK hydrogels. In the absence of KBr, the peak OD of ranPTAK is blue-shifted relative to regPTAK. The red shoulder that is present in both hydrogels (but much more pronounced in regPTAK) is due to delocalized intra- and interchain exciton absorption. Addition of 0.5 M KBr redshifts both spectra, but the red shoulder becomes more prominent only in regPTAK hydrogels, indicating a substantial increase in interchain π-stacking. Changes in OD spectra are accompanied by concomitant changes in PL spectra, which show a salt-induced decrease in the 0–0/0–1 vibronic intensity ratio for regPTAK and a corresponding increase for ranPTAK hydrogels. The 0–0 and 0–1 vibronic peaks are labeled above the PL spectra.

Figure 2A shows that, in the absence of KBr, the OD spectrum of the regPTAK hydrogel is red-shifted and shows a more pronounced shoulder on the red side compared to ranPTAK. This is consistent with the expectation that a lower regioregularity of ranPTAK favors more disordered backbone structures. This in turn leads to a relative decrease in the number density of interchain π-stacking contacts compared to regPTAK. In fact, the shape of the regPTAK spectrum is reminiscent of thin films of poly(3-hexylthiophene), where extensive interchain π-stacking (and resulting semicrystallinity upon thermal annealing) is well-known to take place.^25^ The small-but-discernible red shoulder of ranPTAK at no excess salt appears to diminish further upon addition of salt. In contrast, the amplitude of the pronounced red shoulder of regPTAK increases further in the presence of KBr, suggesting that the number density of interchain excitonic states increases.

Figure 2B shows that the PL spectra of the two CPE hydrogels with no excess salt have similar vibronic 0–0/0–1 ratios *S* ≡ *I*^0–0^/*I*^0–1^, where *I*^0–0^ and *I*^0–1^ are the peak PL intensities of the 0–0 and 0–1 peaks, respectively. The main difference between the two CPEs is additional PL signal on the blue side of the ranPTAK spectrum, consistent with its blue-shifted OD. The fact that the PL spectra of the two CPEs in the absence of KBr are so similar indicates that in both hydrogels emission primarily comes from exciton traps composed of π-stacked interchain domains. Such interchain states display predominantly H-aggregate character, as seen from the fact that *S* < 1.^26−29^ We expect that regPTAK hydrogels have a larger number density of H-aggregate states than ranPTAK based on their OD spectra. Nevertheless, energy-transfer dynamics following photoexcitation in condensed assemblies of conjugated polymers are known to be ultrafast.^27−29^ Evidently most excitons in both hydrogels eventually find similar exciton trap states during their excited-state lifetimes.

Addition of 0.5 M KBr causes *S* to change in the opposite direction for the two CPEs: In the regPTAK hydrogel, *S* drops by ∼10%, whereas it increases by ∼10% for the ranPTAK. We interpret this to mean that the average spatial extent of π-stacked regions increases in regPTAK, corresponding to a net strengthening of interchain interactions. The fact that nominally *S* ∼ 1 for ranPTAK implies that a fraction of the π-stacked regions decouple and give way to spatially separated CPE chains with J-aggregate-like emission.^30,31^

### 3.3 Time-Resolved Photoluminescence Spectroscopy

To get a deeper understanding of the changes in the photophysics with regioregularity and salt, we measured time-resolved PL (TRPL) decays of the gel samples. Deconvolving the instrument response function (IRF) from the measured decay allowed us to compare average PL lifetimes ⟨τ⟩. Given the somewhat unconventional nature of hydrogel samples for TRPL measurements, for a given sample we collected decays at 5 different spots. The spot-to-spot variation was small, showing that on the cm length scale the hydrogels were fairly homogeneous. To account for the small variation, we found ⟨τ⟩ for each spot by using a biexponential model for the decay and then averaged the ⟨τ⟩ values. We use the symbol  to denote this doubly averaged lifetime and *σ*_*R*_ for its standard deviation relative to .

**Figure 3:**
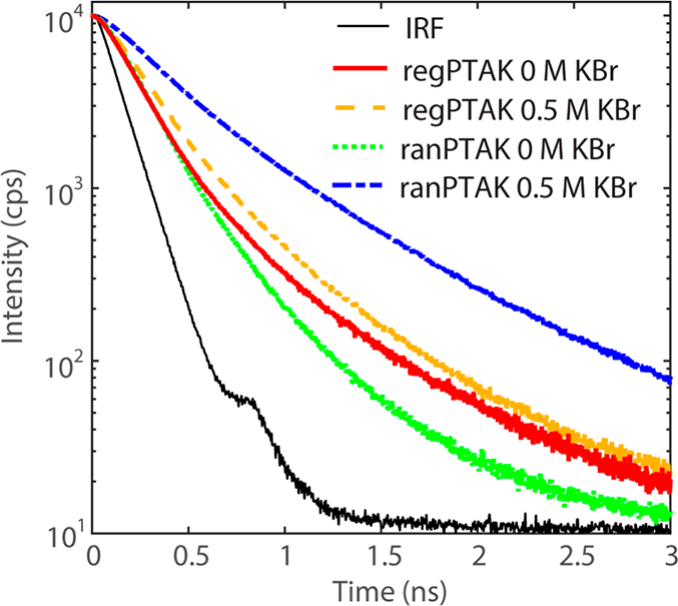
Time-resolved PL decays for PTAK:PDADMAC hydrogels. The legend indicates the concentration of KBr for a given decay curve. Compared to all other samples, the ranPTAK gel with 0.5 M KBr exhibits by far the longest PL lifetime.

Figure 3 shows that the PL decay of the regPTAK:PDADMAC hydrogel changes relatively little upon addition of salt:  increases from 166 ps (*σ*_*R*_ = 0.19) to 198 ps (*σ*_*R*_ = 0.06)—a 19% change. In contrast, the change in the PL decay of ranPTAK from no salt to 0.5 M KBr is substantially larger than regPTAK:  increases from 132 ps (*σ*_*R*_ = 0.08) to 313 ps (*σ*_*R*_ = 0.10), which corresponds to a 137% increase. Increases in PL lifetimes in conjugated polymers are often either due to a breakup of exciton traps associated with π-stacked inter- or intrachain H-aggregate states or a lengthening of the spatial extent of the excitonic wave function along the polymer backbone.^26^

**Figure 4:**
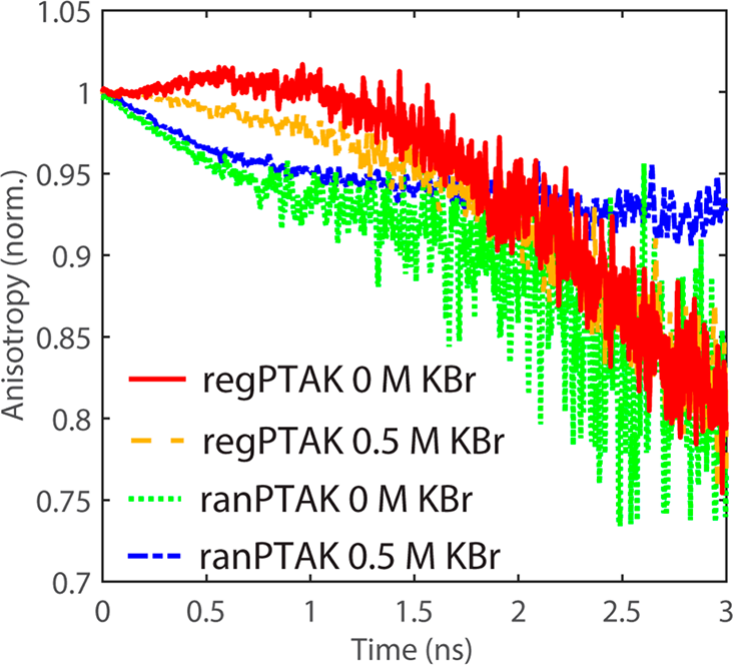
Time-resolved fluorescence anisotropy dynamics of the hydrogels. Unlike regPTAK, ranPTAK hydrogels exhibit a mild decrease in anisotropy over the first 0.5 ns.

To help interpret the observed changes in TRPL, we performed time-resolved PL anisotropy (TRPLA) measurements. The TRPLA is sensitive to the reorientation of the average transition dipole moment of the exciton during its lifetime. Such reorientation can occur either due to motions of the CPE backbone on a time scale comparable to the exciton lifetime, or due to exciton migration between CPE chromophores.^32−36^Figure 4 shows the TRPLA normalized near time zero for the four hydrogel samples. The TRPLA decays for regPTAK gels are relatively weakly time-dependent for the first ∼1.5 ns. In contrast, ranPTAK gels both in the absence and presence of excess salt show a mild but remarkably similar decrease over the first ∼0.5 ns.

We expect rapid motion of CPE chains at early times to be largely arrested in the gel state. Thus, we attribute the initial TRPLA decay of ranPTAK:PDADMAC gels to exciton hopping between ranPTAK chains. Following photoexcitation, ultrafast depolarization due to torsional motions of the CPE backbone precedes the onset of exciton migration.^21^ The part of the depolarizaton associated with exciton hopping on the 100s of ps time scale occurs in the temporal tail end of the exciton diffusion process. Since excitons are preferentially funneled toward lower-energy (lower-bandgap) states, the hopping process at long times must correspond to hopping between relatively extended single chains or proximal π-stacked regions. The fact that the decay of the TRPLA on the 100s of ps time scale is more rapid for ranPTAK than for regPTAK suggests that, on average, the ranPTAK domains are more interconnected than regPTAK domains. This leads to more interchain exciton pathways. The lack of dependence on excess salt of the early time decay in ranPTAK suggests that the primary reason for this observation is due to the local structure of ranPTAK backbone and not the mesoscale structure.

On time scales longer than 1 ns the source of PL depolarization is likely segmental motions of CPE backbones. At first glance it appears that the long-time TRPLA decay of the ranPTAK hydrogel with 0.5 M KBr distinguishes itself by its apparently weaker time dependence. However, we believe that such interpretations must be made with caution as the PL intensity at these times is quite low for all samples but the one with ranPTAK with 0.5 M KBr.

### 3.4 Confocal Photoluminescence Microscopy

**Figure 5:**
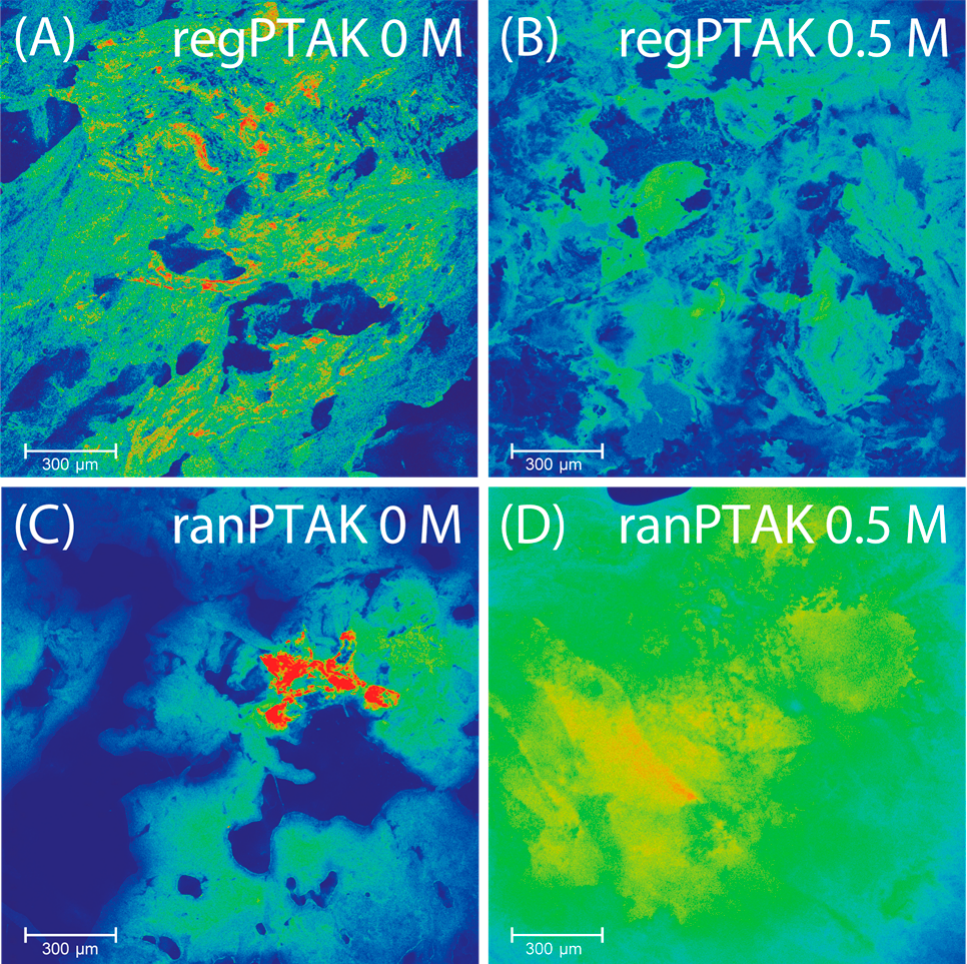
Confocal fluorescence microscope images of PTAK:PDADMAC hydrogels in the absence and presence of excess salt. (A) regPTAK with no KBr; (B) regPTAK with 0.5 M KBr; (C) ranPTAK with no KBr; (D) ranPTAK with 0.5 M KBr. Color corresponds to relative fluorescence intensity within a given image, with hotter colors corresponding to larger intensities.

Are there differences in microstructure that could help explain the rheological and photophysical differences between regPTAK and ranPTAK hydrogels? To help answer this question, we collected confocal PL microscopy images of each gel. Figure 5A,B show representative images for regPTAK:PDADMAC gels with no KBr and with 0.5 M KBr, respectively. regPTAK gels show coarse connective structures on the tens to hundreds of μm scale, with a significant number of PL hot spots distributed throughout the gel. regPTAK:PDADMAC gels with KBr remain fairly coarse, but the relatively high-frequency spatial variations in PL intensity become smoothed out. Evidently, a decrease in the storage modulus of regPTAK gels is associated with a smoothening of regions that gave rise to strongly concentrated PL signal. The net result is a mild increase in  and a significant decrease in *σ*_*R*_.

The ranPTAK gel with no salt (Figure 5C) displays a morphology that is qualitatively similar to the regPTAK gel. Figure 5D shows the ranPTAK:PDADMAC hydrogel in the presence of KBr. The morphological change compared to the gel at no salt is substantial: In the presence of excess salt the gel becomes quite homogeneous with significantly fewer regions with concentrated PL. Evidently this is correlated with a substantially larger  relative to no excess salt.

### 3.5 Electrical Impedance Spectroscopy

**Figure 6:**
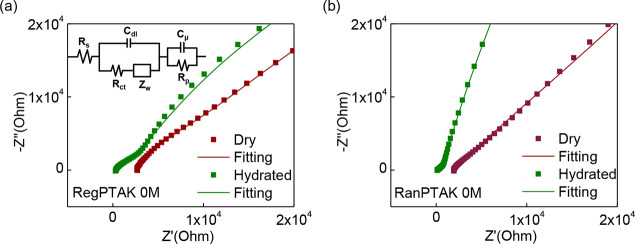
Nyquist plots and fitting curves showing the real (*Z*′) and imaginary (*Z*″) impedance for (A) regPTAK:PDADMAC and (B) ranPTAK:PDADMAC hydrogels with no excess salt in dry and hydrated state at 298 K in the frequency range between 0.1 Hz and 0.1 MHz. The nonzero impedance of the dried hydrogels shows that they are electron (or hole) conductors, and the improvement of conductivity in the hydrated state indicates that the samples are also ionic conductors. (Inset) Equivalent circuit model used to fit the EIS response.

Although we have unraveled the influence of regioregularity on the photophysics, it is less clear how differences in hydrogel microstructure manifest in their charge transport properties. To answer this question, we performed electrochemical impedance spectroscopy (EIS) measurements on both dry and hydrated (90% relative humidity) hydrogels in the absence of excess KBr. The results of these measurements are shown as Nyquist plots in Figure 6. Figure 6A,B implies that both regPTAK:PDADMAC and ranPTAK:PDADMAC gels, respectively, are conductive in their dry states.

In the dehydrated state, we expect vanishing ionic charge transport. This is because the dielectric constant of carbon-based materials (∼3–4) is sufficiently low that the electrostatic binding energy of two oppositely charged ions near their equilibrium separation is much larger than thermal energy at room temperature. This leads to a large activation barrier for ion hopping. Thus, the residual conductivity is most likely due to electronic charge carriers.^26,27^ This is not unexpected, since it is well-established that gold electrodes can readily inject holes into the polythiophene valence band given the favorable alignment between the gold Fermi level and the HOMO level of the conjugated polymer.^37^

Upon hydration, the conductivity of both hydrogels increased, implying an additional transport contribution from mobile small ions. In the hydrated state of hydrogel samples with no excess salt, we believe the ionic conductivity primarily comes from two sources: (a) the counterions released upon formation of the interpolyelectrolyte complex and (b) incomplete interpolyelectrolyte complexation, leading to small ions bound to uncomplexed polyelectrolyte chains. Thus, in these samples the ionic charge carriers are hydrated K^+^ and Cl^–^ ions.

To get a deeper understanding of the change in the charge transport upon hydration for the two hydrogels, we modeled the EIS data using the commonly used Randles model with an equivalent-circuit shown in the inset of Figure 6A.^38^ This model has successfully been applied to understand the EIS response of similar hydrogel samples.^39,40^ The fitting parameters included the electrolyte resistance, *R*_*s*_, the transfer resistance, *R*_*ct*_, the double layer capacitance in the electrolyte, *C*_*dl*_, the Warburg resistance, *Z*_*w*_, the chemical or space charge capacitance, *C*_*μ*_, and the transport resistance, *R*_*t*_.^40^*R*_*s*_ is determined by the interaction between the electrolyte and the hydrogel: the higher the ionic concentration and the carrier mobility in the hydrogels, the lower the *R*_*s*_. The dried hydrogels have *R*_*s*_ values of 2574 Ω (regPTAK) and 281.2 Ω (ranPTAK), while the hydrated hydrogels have relatively low values of 281.2 Ω (regPTAK) and 12.5 Ω (ranPTAK), respectively, due to the ionic transport. Figure S1 of the Supporting Information displays impedance curves in the hydrated state for each hydrogel in triplicate, showing that the conductive behaviors of both hydrogels are representative. Therefore, we infer that PTAK:PDADMAC hydrogels are mixed electronic/ionic conductors.^7^ However, it is certainly the case that the injected number density of electronic charge carriers is significantly smaller than the equilibrium electron or hole density would be had the CPE backbone been chemically doped.

## 4 Discussion

We have shown that, in the absence of excess KBr, differences in photophysical properties between regPTAK and ranPTAK hydrogels are unremarkable. Steady-state OD and PL spectra are both consistent with a significant fraction of π-stacked domains. The primary difference can be traced to in the size of chain regions over which H-aggregate excitons are coherently delocalized, which naturally arises given the difference in regioregularity. Such a difference is expected for regioregular versus regiorandom polymers, as demonstrated previously via poly(3-hexylthiophene) thin films. In such films, differences in regioregularity can give rise to large differences in the charge mobility.^41^ However, the charge mobility measures the transport of charge through the entire thin film. In contrast, the spectroscopic response reflects local excitonic states. Additionally, the environment in a thin film differs drastically from that of the hydrogel, where hydration and electrostatic interactions are of substantial importance. The relatively small difference in  in the absence of excess salt is consistent with our steady-state spectroscopy results.

How can we understand the fact that  upon addition of KBr was significantly larger for ranPTAK hydrogels than for regPTAK ones? We believe that significant hints are provided by the smaller *G′* and the smoother morphology of ranPTAK relative to regPTAK. The fact that *G′* decreased for both gels upon addition of KBr suggests that the excess small ions either (i) lower the strength of electrostatic interactions between PTAK and PDADMAC chains or (ii) infiltrate π-stacked regions between proximal PTAK chains (or regions of the same CPE chain). We previously showed that, in dilute solution, both regPTAK and ranPTAK complexed with the same cationic CPE in a similar average conformation.^42^ It is tempting to conclude that there should not be a large difference between the electrostatic interaction of regPTAK and ranPTAK with PDADMAC. However, the average local environment proximal to a given complexed PTAK:PDADMAC region may differ from that of an inter-CPE complex in dilute solution. Thus, the possibility that the backbone regioregularity of the CPE modulates the mean electrostatic interaction strength and its higher moments within the hydrogel cannot be dismissed.

Nevertheless, we believe that the more likely reason for the difference in  upon addition of salt is due to the influence of small ions on π-stacked PTAK regions with different regioregularities. In regPTAK, the significantly larger regioregularity allows for effective chain packing and formation of relatively ordered domains, as clearly seen in the difference in OD spectra. The OD signatures that we observe have previously been strongly correlated with the appearance of long-range order.^25,43,44^ In contrast, the low regioregularity of ranPTAK leads to fewer ordered domains and thus poorer packing. We speculate that this in turn likely allows the small ions to readily penetrate voids between π-electron densities.

When the small ions enter the gel structure, they likely swell and push apart weakly π-stacked ranPTAK chains. The net result is a large relative increase in the PL lifetime as a significant fraction of interchain H-aggregate excitons is converted to J-aggregate-like states that are largely delocalized along single CPE chains. On average, we expect that this would lead to a more spatially homogeneous network with a narrower distribution of interchain π-stacking interaction strengths, centered about a mean that is smaller than in the absence of excess salt.

It is interesting that (i) only ranPTAK hydrogels displayed a short-time TRPLA decay and that (ii) this decay component for ranPTAK was unchanged in the absence and presence of KBr. Since the OD and PL spectra of ranPTAK hydrogels underwent substantial changes upon addition of KBr, observation (ii) implies that this TRPLA decay component does not reflect differences in the strength of π-stacking interactions within domains containing multiple interacting CPE chains. Thus, we propose that the fast TRPLA component reflects exciton transfer along ranPTAK tie chains that connect π-stacked domains. Together with observation (i), this would imply that the number density of such tie chains is significantly larger in ranPTAK hydrogels. Within this model, in regPTAK gels, the significantly fewer tie chains must connect more aggregated π-stacked domains with a diminished ⟨τ⟩. The fact that the ranPTAK hydrogel has a significantly lower viscosity than regPTAK likely reflects the fact that the activation energy needed to rearrange weakly bound π-stacked domains is smaller than when the domains are extended and strongly π-stacked.

We believe that the structural model that we propose based on photophysical and microscopy measurements is also consistent with the EIS measurements. We found that *R*_*s*_ was significantly smaller for ranPTAK than for regPTAK, both in the dry and hydrated states. We argue that similar structural characteristics that lead to more facile exciton hopping at long times in ranPTAK hydrogels likely correlate with increased rates of ion transport. That is, a larger tie chain density and fewer aggregated domains likely give rise to a more open ranPTAK chain structure, thereby lowering the average barrier to ion migration relative to regPTAK.

## 5 Conclusion

In summary, our work shows that both the mechanical response and the optoelectronic properties of hydrogels formed via electrostatic interpolyelectrolyte complexation can be straightforwardly varied by varying the ratio of regioregular to regiorandom domains along the CPE backbone. We also showed that such hydrogels display salt-tunable storage moduli that are significantly larger in the low-salt limit than hydrogels formed from pure CPEs and are more stable against dissolution in common polar (and nonpolar) organic solvents. Finally, we demonstrated that these gels can act as mixed electronic/ionic conductors. We believe that the relative ease with which such optoelectronically active, stable, and mechanically tunable hydrogels can be produced makes them intriguing candidates for biomedical and sensing applications. We can also envision that such an optoelectronic hydrogel could serve as a kind of matrix which could be infiltrated with additional molecular components to build a larger system capable of electronic communication. One criticism that may be levied against these systems is that it takes a secondary electronically inactive component to form the hydrogel, perhaps thereby limiting the total charge transport that could in principle be achieved in these materials. However, it has been previously shown that conjugated polymers can exhibit impressively large charge mobilities even at very high dilution in a solid matrix composed of an electronically inactive polymer.^28^ It must be pointed out that the total electrical conductivity of the hydrogel would be a product of the carrier mobility and the carrier density. Dilution of the conjugated polyelectrolyte component would tend to lower the carrier density. Nevertheless, we believe that the need for an oppositely charged, nonconjugated polyelectrolyte is not a major limitation and, in fact, could be viewed as another variable that can be further manipulated to optimize hydrogel properties.

## Supporting Information Available

Replicate Nyquist plots of hydrogel samples and PL anisotropy decays with errors bars (PDF)
