# Supplementary material for: Influence of Backbone Regioregularity on the Optoelectronic and Mechanical Response of Conjugated Polyelectrolyte-Based Hydrogels
Source: J Phys Chem B. 2023 Mar 7;127(10):2277–85. doi: 10.1021/acs.jpcb.3c00152 (PMC10026064; doi:10.1021/acs.jpcb.3c00152)
Supplement: Supplementary file 1 — jp3c00152_si_001.pdf [file jp3c00152_si_001.pdf]

Supporting Information

for

Influence of Backbone Regioregularity on the Optoelectronic and Mechanical Response of Conjugated Polyelectrolyte-Based Hydrogels

William R. Hollingsworth,[1] Anna R. Johnston,[1] Manping Jia,[2] Le Luo,[2] Yunjeong Park,[2] Walter Meier,[1] Jack Palmer,[1] Marco Rolandi,[2] Alexander L. Ayzner[1]*

Department of Chemistry and Biochemistry, University of California Santa Cruz, Santa Cruz, CA, 95064, USA

[2] Electrical and Computer Engineering Department, University of California Santa Cruz, Santa Cruz, CA, 95064, USA

*aayzner@ucsc.edu

S1. Electrochemical Impedance Measurements

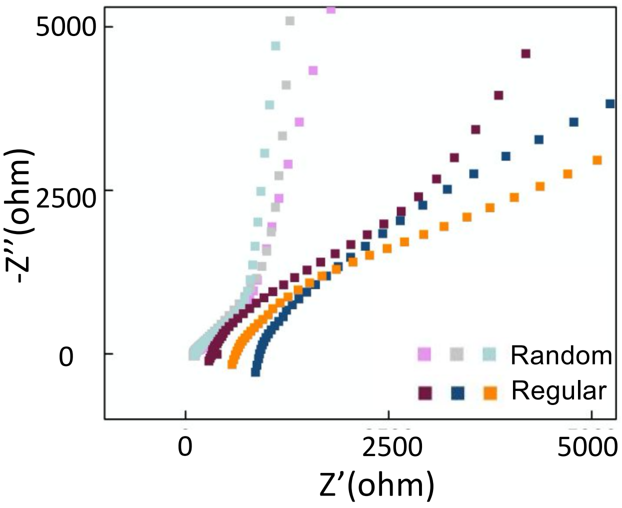

Figure S1. Nyquist plots of three hydrated rePTAK and raPTAK hydrogel samples with no excess KBr.

The slope of the impedance curve for the raPTAK:PDADMAC hydrogel in the hydrated state is larger than that of rePTAK. Considering that the structure of raPTAK hydrogel is more homogenous with highly connected domains, the reason for this difference could be that ion diffusion here is faster than that in strongly aggregated rePTAK hydrogels, so raPTAK hydrogel presents more capacitive behavior.[1]

S2. PL Anisotropy with Error Bars

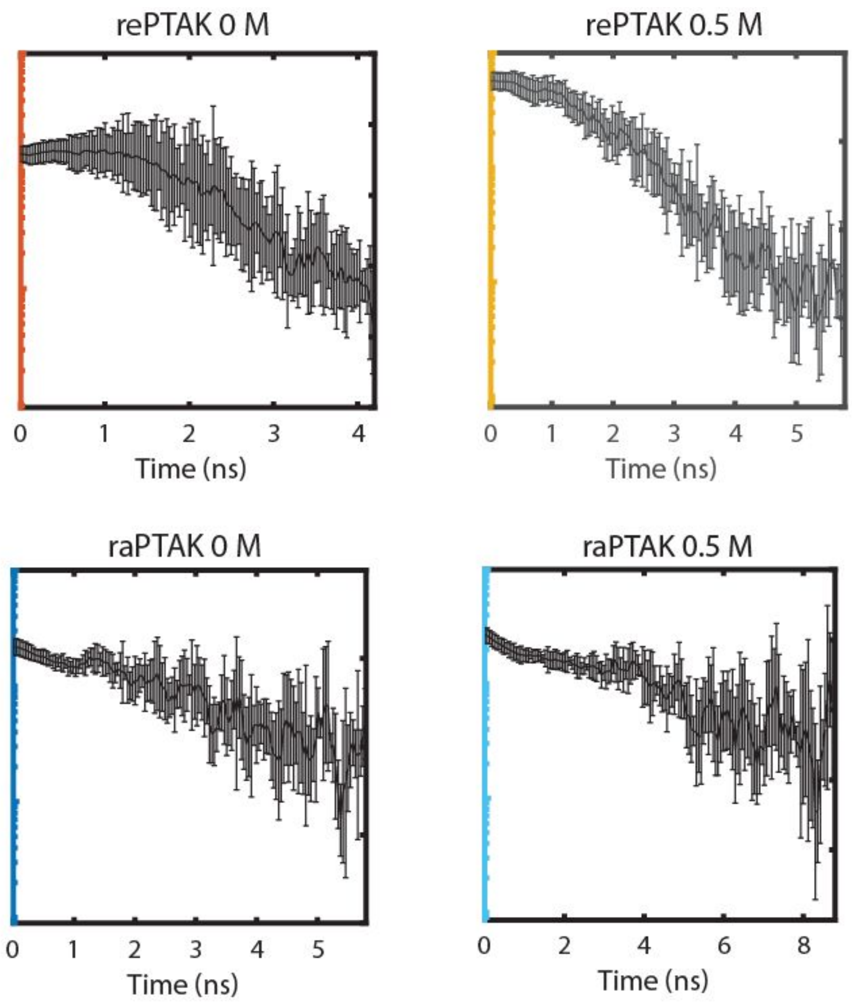

Figure S2. PL anisotropy dynamics for the hydrogels illustrating the error bars generated by averaging anisotropy decay curves collected at 5 different positions of a given sample. The sample and the KBr concentration are labeled above each corresponding panel.

REFERENCES

1. Xie, J.; Yang, P.; Wang, Y.; Qi, T.; Lei, Y.; Li, C. M., Puzzles and Confusions in Supercapacitor and Battery: Theory and Solutions. *Journal of Power Sources* **2018**, *401*, 213-223.
